# Junctions, Transporters, and Interactions of Endothelial Cells: Regulation by Ethanol

**DOI:** 10.3390/ijms27062695

**Published:** 2026-03-16

**Authors:** Chitra D. Mandyam, Angelica Vandekerkhoff, Sehwa Jung, Dhwani Kharidia, Igor Ponomarev, Brent Kisby

**Affiliations:** 1 VA San Diego Healthcare System, San Diego, CA 92161, USA; avandekerkhoff@health.ucsd.edu (A.V.); sejung@ucsd.edu (S.J.); dkharidia@ucsd.edu (D.K.); 2 Department of Anesthesiology, University of California San Diego, San Diego, CA 92093, USA; 3 Department of Pharmacology and Neuroscience, Texas Tech University Health Sciences Center, Lubbock, TX 79430, USA; igor.ponomarev@ttuhsc.edu; 4 Center for Translational Neuroscience and Therapeutics, Texas Tech University Health Sciences Center, Lubbock, TX 79430, USA

**Keywords:** alcohol, blood–brain barrier, vascular proteins, gene expression, sex differences

## Abstract

Alcohol (ethanol, an intoxicating agent in all alcoholic beverages) is the most widely consumed beverage in the United States and is a leading risk-factor for cerebrovascular diseases. Although neurons, microglia, and astrocytes have been moderately studied for their responsiveness to ethanol, the brain vasculature is minimally explored and is emerging as a key player in the interplay between neuroinflammation, cerebrovascular disease, and alcohol use disorder (AUD). The blood–brain barrier (BBB), a critical regulator of brain homeostasis, relies on the coordinated function of various cellular and molecular components to maintain its immune-privileged status. Emerging evidence indicates that chronic ethanol exposure disrupts BBB function, contributes to neurovascular dysfunction, and increases brain permeability to peripheral immune factors. This review introduces the endothelial cells (ECs) that make up the BBB and provides a brief overview of the junction proteins and transporters that assist with EC function and EC interactions with other cells of the neurovascular unit, including pericytes, smooth muscle cells, and perivascular macrophages and glial cells. In addition, this review highlights ethanol’s effects on ECs and the cells that interact with them. Lastly, given the mounting evidence on gender differences in AUD and the supporting sex differences in ethanol consumption in preclinical models, this review discusses the discovered sex differences in EC-specific genes and identifies open questions such as the influence of sex-dependent differences, genetic factors, and their interactions with ethanol on EC function. Taken together, a deeper understanding of how ethanol disrupts EC structure and function will advance therapeutic strategies to mitigate neuroinflammation and related pathologies associated with chronic ethanol exposure.

## 1. Introduction

The blood–brain barrier (BBB) is fundamental in maintaining homeostasis in the central nervous system (CNS), particularly in the brain, and consequently, BBB disruption has been implicated in various neurodegenerative and neurological disorders, including Alzheimer’s disease, Parkinson’s disease and ischemic stroke [1,2,3]. Brain endothelial cells (ECs) constitute the core component of the BBB. Therefore, while the EC’s restrictive nature protects the brain from harmful pathogens, it also limits delivery of potential therapeutic drugs, acting as a major barrier for treatments of said neurodegenerative and neurological diseases. As a result, there is a growing need to develop models to study the BBB, specifically the ECs and its vascular counterparts, to not only better understand its function and disruption, but to also advance efforts to deliver therapeutic drugs for brain diseases [4].

Alcohol use disorder (AUD) is a chronic relapsing brain disease that occurs with long-term alcohol consumption. AUD represents a major global health burden, affecting millions of individuals in the United States [5]. Among the most profound and lasting consequences of chronic ethanol exposure are the neurological complications, including cognitive impairment, neurodegeneration, and increased susceptibility to cerebrovascular accidents [6,7,8]. While the direct neurotoxic effects of ethanol on other cell types has been repeatedly studied [9,10,11,12], mounting evidence suggests that ethanol’s impact on the brain’s vascular cell types may be equally critical in mediating alcohol-related neurological dysfunction [13].

The molecular basis of ethanol-induced cerebrovascular dysfunction involves widespread alterations in gene expression across different vascular cell populations [14,15,16]. ECs respond to ethanol exposure through the dysregulation of genes and proteins controlling tight junction integrity, transport systems, and barrier permeability [13,17,18]. These transcriptional and translational changes compromise the protective barrier function that is essential for normal brain homeostasis. Recent advances in single-cell genomics and transcriptomic profiling have begun to reveal the complexity of ethanol-responsive gene networks within individual vascular cell types [19]. However, our understanding of how these cell-specific responses integrate to produce the overall pathophysiology of alcohol-related cerebrovascular dysfunction remains incomplete. Furthermore, the temporal dynamics of gene expression changes and the potential for reversibility of ethanol-induced alterations are poorly understood.

This review aims to introduce the BBB and ECs that make up the BBB and provide a brief overview of the vascular network supporting the BBB including, brain microvascular ECs and their interactions with pericytes, smooth muscle cells, and perivascular macrophages and glial cells. In addition, the following sections will incorporate the interactions between the brain’s vasculature network and ethanol based on limited publications, specifically examining molecular and cell type-specific alterations because of ethanol exposure. Lastly, given the mounting evidence on gender differences in AUD and the supporting sex differences in ethanol consumption in preclinical models, this review discusses the discovered sex differences in EC specific genes and identifies open questions such as the influence of sex-dependent differences, genetic factors, and their interactions with ethanol on EC function. By integrating established knowledge of cell-specific ethanol effects with emerging transcriptomic insights, this review aims to provide a comprehensive framework for understanding ethanol-induced cerebrovascular dysfunction while addressing future directions.

## 2. The Neurovascular Unit of the BBB

**Figure 1:**
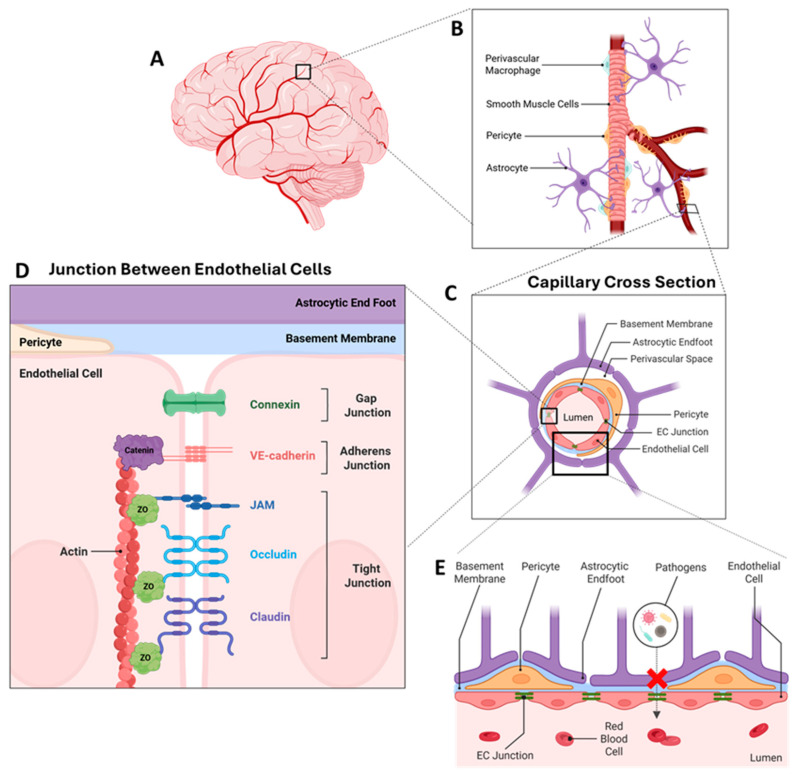
Schematic of the brain vascular system in a normal health brain. (**A**) A cartoon representation of the human brain with capillaries. (**B**) The brain vascular system is magnified to indicate the presence of the components of the neurovascular niche, including astrocytes, pericytes, smooth muscle cells and perivascular macrophages. (**C**) The cross section of a capillary is shown to indicate how endothelial cells are surrounded by astrocytic endfeet and pericytes to form closed communication within the vascular niche. (**D**) The endothelial junction is magnified to display the various junctional proteins that form the blood–brain barrier. (**E**) Endothelial cells and the surrounding vascular niche are magnified to indicate the tightness of the junction between the cells and the inhibition of pathogens into the lumen protecting the brain from the peripheral infiltration of toxic molecules.

**Figure 2:**
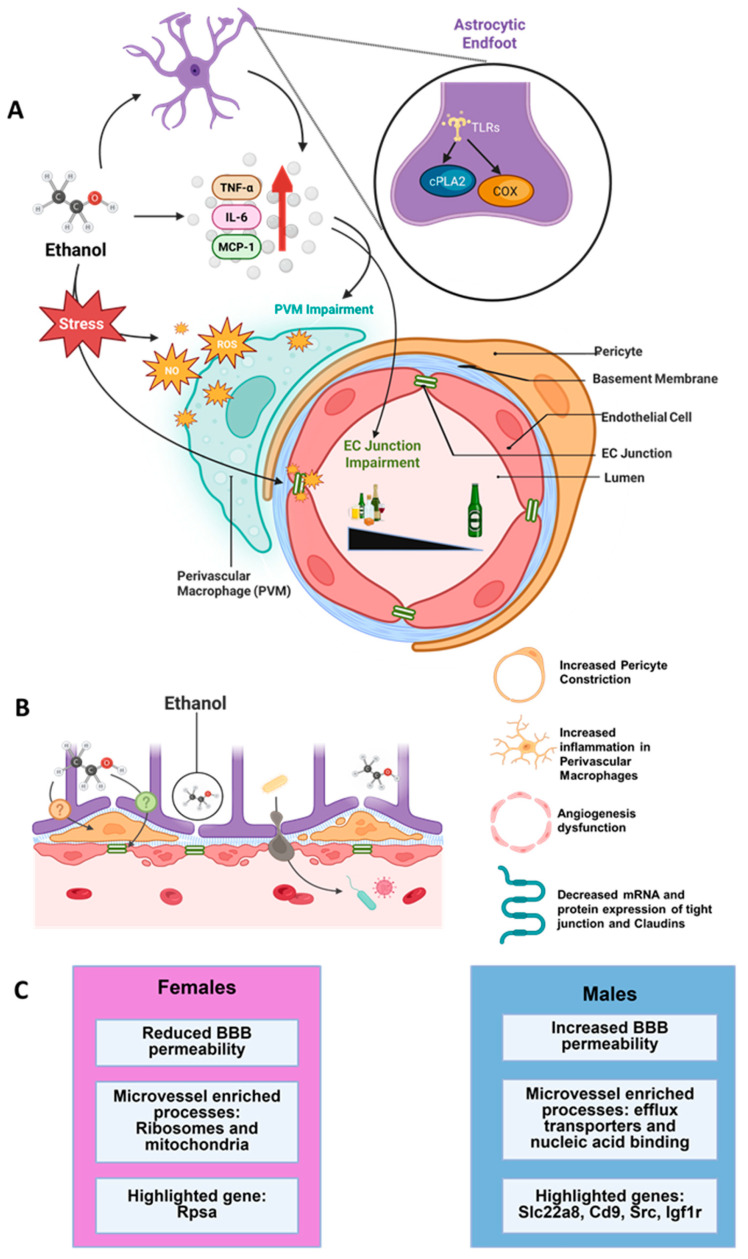
Schematic indicating the mechanistic details on the ethanol modulation of the neurovascular niche (**A**) and the blood–brain barrier (BBB); (**B**,**C**) in pre-clinical models of ethanol exposure, ethanol differentially alters the permeability of the BBB in female and male rodents with significant differences in the endothelial genes altered by ethanol exposure.

The BBB’s special properties are made possible by the formation of the regulated neurovascular unit (NVU), comprising ECs, mural cells (pericytes, smooth muscle cells), glia (astrocytes, microglia, oligodendrocytes), and neurons, all of which work in unison to regulate the BBB and protect the brain (Figure 1A–C; [2,20,21,22]). NVUs at arterioles, small arteries, and veins have ECs making up the innermost layer of the vessel, with the basement membrane separating ECs from one to three layers of smooth muscle cells (SMCs). However, capillary NVUs have ECs and pericytes sharing a basement membrane, with approximately one pericyte, tightly surrounding every two to four ECs [1,23]. Arteriolar and capillary NVUs are enclosed by astrocytic endfeet, however NVUs at penetrating arteries have SMCs enveloped by pia, with the Virchow–Robin space between the pia and endfeet [1]. Although all these cells in the NVU contribute to the BBB’s functioning, ECs are the primary cells responsible for the BBB’s restrictive permeability [24,25]. In the following sections we will discuss the basic function of ECs, the proteins that moderate and maintain the junction between ECs, the transporters that assist with EC function and cell–cell interaction between ECs and other cell types of the NVU. In addition, we will discuss the limited findings on ethanol’s effect on ECs, and the discovered sex differences in EC specific genes and identify open questions such as the influence of sex-dependent differences, genetic factors, and their interactions with ethanol on EC function.

## 3. ECs and Basic Functions of ECs

ECs are the primary cell type that line the lumen of the blood vessels throughout the body. Notably, ECs of the brain have consistently demonstrated significant divergence from ECs of other tissues, highlighting the heterogeneity of the endothelial transcriptome and translatome between the brain and the body [26,27,28,29,30]. In addition, the permeability of brain ECs are highly restrictive in that ECs in the brain have significantly decreased permeability compared to ECs in the body [31,32]. To add to the divergence, there are several properties that set brain ECs apart from those in the periphery, ultimately contributing to the BBB’s selective nature: (1) They have junctional complexes which hold brain ECs together, which greatly limits the flow of polar ions and solutes compared to peripheral ECs [2,33,34,35]; (2) there are greatly decreased rates of vesicular trafficking and transcytosis [20]; (3) they have higher mitochondria content, necessary for the high ATP requirements of the brain [36]; (4) nutrient delivery and uptake is exclusively mediated through plasma membrane transporters [37]. Taken together, although having varying degrees of permeability, capillary endothelium generally prevents the entry of large molecules and proteins, while allowing the free flow of ions and sugars.

## 4. ECs Are Held Together by Junctional Complexes

Brain ECs comprise both active and passive barrier phenotypes. Junctional complexes between brain ECs are comprised of tight, adherens and gap junctions, and play a unique role in maintaining BBB permeability (Figure 1D,E). Tight junction proteins limit the paracellular movement of molecules and contribute to the passive properties of the BBB. Tight and adherens junctions together form the inter-endothelial junction, adhering ECs together and ensuring barrier function [38]. Below we will provide information on each of these junction complexes and their role in maintaining a healthy BBB and discuss the limited information on ethanol’s effect on these proteins.

### 4.1. Gap Junctions

Gap junctions (GJs) are composed of six connexins to form connexons (hemichannels) in the EC’s plasma membranes, and are essential in intercellular communication, as ions and small molecules pass through these GJs and relay signals to neighboring cells [39,40]. With freeze-fracture images, GJs present as intramembrane particles clustered with central depressions around the channel pore [41]. Unlike tight junctions and adherens junctions, the goal of GJs is not to form the tightest seal possible between cells, but to form channels connecting the cytoplasm of ECs [42]. Connexin 37, connexin 40, and connexin 43 (Cx37, Cx40, and Cx43) are known to be expressed in ECs at varying amounts depending on the type of vasculature [42,43]. Although blocking connexin channels did not affect the formation of tight junction strands, the function of tight junctions decreased by over 19% after blocking channels to Cx40 and 43 [44]. Additionally, Cx40 and 43 colocalized with tight junction molecules, occludin, claudin-5, and ZO-1 [44], confirming the functional significance of connexins in barrier effectiveness.

### 4.2. Tight Junctions

Compared to ECs in peripheral capillaries, those in the CNS are 50–100 times closer, resulting in the extremely high transepithelial/transendothelial electrical resistance (TEER) of blood vessels (a quantitative measure of endothelial function), in addition to limited transmission of solutes throughout the BBB [20]. Using ultrastructural electron microscopy analysis, brain ECs’ tight junctions (TJs) were characterized by Reese and Karnovsky after they administered horseradish peroxidase (HRP) injections in mice [25]. Their study revealed that HRP was unable to permeate through TJs between ECs, citing the “barrier” of the BBB as the monolayer of ECs connected by TJs. They also found that some of the HRP was pinocytosed, but were not transported to the abluminal membrane, and were instead deposited into the basement membrane. Located on the most apical side of the membrane, TJs primarily consist of claudins, occludins, zonula occludens, and junctional adhesion molecules. Together, these TJ proteins tightly regulate paracellular solute and ion diffusion, resulting in a high TEER of >1500 Ohm × cm^2^ [38,45,46,47]. This is much higher compared to the TEER of peripheral vessels in the intestines and muscles, which ranges from 2 to 20 Ohm × cm^2^ [48,49]. We will further discuss the TJ proteins zonula occludens, occludins and claudins, and highlight their role in maintaining BBB integrity.

Zonula Occludens: Zonula occludens (ZO) are a family of cytoplasmic scaffolding proteins that constitute TJs [50,51]. ZOs are peripheral membrane proteins localized in the immediate vicinity of the plasma membrane of TJs in epithelial cells and ECs [52]. ZOs are also found in cells that lack TJs, such as fibroblasts and cardiac muscle cells, where they colocalize with cadherins [53]. Three ZO proteins (ZO-1, ZO-2, and ZO-3) have been identified to date [54]. They are cytoplasmic proteins and members of the large family of membrane-associated guanylate kinase proteins and are required for regulation and maintenance of TJ structure. They form a complex on the cytoplasmic side of TJ, and other TJ proteins bind to the N-terminal half region of ZO proteins [55]. ZOs initiate and facilitate polymerization of claudin to form TJ strands [56]. In vitro studies indicate that in ZO-1 knockout and ZO-2 deficient cells, TJs entirely failed to form, but were able to reform TJs after exogenous expression of ZO-1 and -2, demonstrating the mechanistic role of ZOs in TJ function [56]. Taken together, the functional significance of ZOs in TJ structure and function is quite notable and appears to be important in maintaining TJ stability [56,57].

Occludins: Occludins are 65 kDa proteins, which were first found to be exclusively located at TJs [58]. cDNA sequencing additionally revealed that occludins contain four transmembrane domains and five cytoplasmic domains [58]. Occludins localize at TJs by directly associating with ZO-1, through occludin’s domain E [59]. With immunofluorescence and electron microscopy, occludin has been found to be expressed in TJs, however they are expressed at slightly lower rates than ZO-1, suggesting that ZO-1 is necessary for occludin’s localization at TJs [60]. Occludins’ structure and hydrophilicity plot resembled that of connexins, an integral protein in GJs [58]. Additionally, the overexpression of occludin in insect cells resulted in formations of short TJ-like strands [61]. This, along with their similarity to connexins, originally suggested that occludins were necessary for TJ formation and function. However, it was later observed that functional TJ strands formed in occludin-deficient mice, with no significant differences between wild-type and occludin-deficient groups, indicating the existence of another unidentified major TJ protein [62]. Interestingly, occludin-knockdown mice have demonstrated some defects, including the development of calcium deposits along small vessels, hearing loss, and male infertility [63,64]. This may indicate that occludins are an important regulatory protein, rather than a structural protein for TJs.

Claudins: Claudins are a family of proteins that act as the primary constituent for TJs in both ECs and epithelial cells [65]. Like occludins, they contain four transmembrane domains but have a distinct transcriptional profile from occludins [66]. To date, there are a total of 28 known proteins making up the claudin family in mammals [67]. Immunofluorescence has demonstrated that specific claudin proteins comprise differing barriers in various tissues and organ systems: Claudin-11 in oligodendrocytes myelin sheets, claudin-2 in pancreatic epithelia, claudin-3 in liver hepatoctyes, and claudin-1 in lung bronchioles [65,68,69,70]. In the brain, claudin-1, -3, -5, and -12 have been found in TJs at ECs [71,72]. It has been determined that claudins are a major structural component of TJs, with studies demonstrating that claudin-1 and -2 form TJ-like strands when introduced in fibroblasts lacking TJs [66]. Claudin-5 was found to be an exclusively endothelial-specific protein, consolidated at the cell–cell contact regions of ECs; as such, it is a major TJ protein in CNS ECs, with claudin-5 mRNA levels expressed over 100 times more than Claudin-12 [73,74]. Claudin-5 deficient mice also demonstrated a “leakier” BBB, resulting in a size-selective loosening and increased BBB permeability for small molecules < 800 D [72]. Taken together, the functional significance of claudins in TJ structure and function is quite notable and appears to be important in maintaining TJ stability [75].

### 4.3. Tricellular Contacts

There are specialized TJs at the contact point of three cells to ensure a sealed barrier, with a narrow tube formed in the extracellular space of tricellular contacts (TCs) [76]. Tricellulin and angulin proteins have been identified as major TC components, with angulin recruiting tricellulin to TC sites, and the depletion of tricellulin and angulin resulting in a lack of TJs at TCs [76]. Notably, tricellulin or angulin were not found in ECs of other types of tissues, including pancreas, aorta, and kidney tissue, which may suggest the importance of TJs at TCs to the BBB’s restrictive properties [76].

### 4.4. Junctional Adhesion Molecules

Junctional adhesion molecules (JAMs) are a family of immunoglobulin proteins present at the apical regions of TJs, promoting homophilic adhesion and regulating monocytic transmigration [77]. Both cis- and trans-dimerisation mediate the function of JAM-A, which ultimately contributes to TJ integrity [78,79,80].

### 4.5. Adherens Junctions

On the basolateral side of the membrane, adherens junctions (AJs) are made up of vascular endothelial cadherin (VE-cadherin or cadherin-5, Cdh5) proteins and primarily support the formation and maintenance of TJs, as well as adhesive cell–cell interactions [46]. Catenins are cytoplasmic plaque proteins that anchor Cdh5 to the actin cytoskeleton, similar to ZO-1 and -2 in TJs [46]. α-catenin, β-catenin, and Cdh5 were found at cell–cell contact areas across all cells, regardless of confluency [81]. However, γ-catenin (plakoglobin) was only present in areas with tightly confluent monolayers, along with an increase in its mRNA levels [81]. Notably, α-catenin, β-catenin, and Cdh5 were all associated with one another in the early stages of cell adhesion. But plakoglobin is only associated with junctions when cells approached maturity, confluence, and were cytoskeleton bound [81]. Necessary for BBB integrity, the depletion of β-catenin in mice resulted in increased vasculature permeability, seizures, and hemorrhages [82]. AJs and TJs are interconnected, as Cdh5 indirectly upregulates claudin-5 expression by phosphorylating and inhibiting FOXO1 [83].

Cdh5 is an EC-specific transmembrane AJ protein [84]. The *CDH5* gene is located in the long arm of chromosome 16 in humans [85] and chromosome 19 [86] in rats. The restricted cell specificity of Cdh5 indicates that it is a critical component of the BBB. For example, in naïve mice, loss-of-function studies show that Cdh5 is necessary for embryonic angiogenesis, and partial deletion studies show that Cdh5 regulates junctional integrity in adulthood [87,88]. In mice models of inflammatory disease states, mechanistic studies show that inflammatory cytokines alter the activity and expression of Cdh5 which leads to the dissociation of Cdh5 junction complexes in endothelial cells and result in reduced BBB permeability and leakage of cytokines and leukocytes [89,90]. Findings from human studies demonstrate that patients with diseases related to BBB dysfunction, such as ischemic stroke and coronary artery disease, have increased circulating levels of Cdh5, suggesting that levels of Cdh5 could be used as a potential biomarker in certain clinical settings [91,92].

In the context of inflammatory responses, in vitro studies show that pro-inflammatory cytokines and other inflammatory mediators downregulate Cdh5 expression and increase vascular permeability [93,94,95]. Similarly, inflammatory cytokine-induced reduction in Cdh5 expression is observed in animal models of systemic inflammation and sepsis [96]. Additional in vitro studies show that the increased expression of Cdh5 occurs as a consequence of reduced activity of nuclear factor kappa B (NF-κB; a transcription factor that regulates expression of cytokines) [97], and additionally, pro-inflammatory cytokine-induced reduction in Cdh5 expression is dependent on NF-κB activity [98]. Taken together, the functional significance of Cdh5 in AJ structure and function is quite notable and appears to be important in maintaining BBB stability and regulating neuroimmune responses in vitro.

### 4.6. Ethanol Modulation of Junctional Proteins

As discussed above, junction proteins of the ECs protect the brain from molecules that are impermeable at the BBB, and therefore, the disruption of junction proteins permit typically impermeable molecules to reach the brain parenchyma [99,100]. Additionally, the BBB ECs exhibit active efflux that limit trans-cellular transport. In vitro studies using primary human brain microvascular endothelial models have demonstrated that ethanol exposure increases permeability and reduces barrier integrity [101]. Specifically, studies in endothelial cell models have shown that moderate to high ethanol concentrations (100 mM and higher) reduced the expression of TJ proteins ZO-1, occludin and claudin-5 [102,103,104]. Notably, the ethanol-induced reduction in these TJ proteins was mechanistically driven by the activation of protein kinase C and reactive oxygen species [103,104], indicating a role for ethanol-induced oxidative stress in BBB disruption (Figure 2). Supporting the in vitro findings, studies from animal models of moderate to severe alcohol use disorder showed reduced the expression of ZO-1, occludin, and claudin-5 in the prefrontal and occipital cortices [18,105] and ECs of the entire brain [106] after weeks of ethanol consumption. With respect to JAMs, although no studies have evaluated the effect of ethanol on JAMs in brain tissue, studies from in vitro models of intestinal cells demonstrate that ethanol reduces the expression of JAM-A and disturbs the integrity of intestinal cell barriers [107]. Ethanol’s effect on Cdh5 is less studied, and in vitro data from endothelial cell models indicates that ethanol induced endocytosis of Cdh5 occurs at higher doses and could play a role in cancer metastasis [108]. In conclusion, these studies suggest that high-dose or long-term ethanol consumption could diminish the BBB and contribute to the development or exacerbation of neurological conditions associated with BBB disruption.

## 5. Transporters Assist with the Critical Functions of the ECs

In the CNS, vasculature is equipped with two major transport systems: The vesicular transcytosis and protein-mediated transport. The vesicular transcytosis system functions in the larger veins and arteries of the CNS and is mediated by clathrin- and caveolae-mediated transcytosis [109]. Much of the CNS vasculature is, however, capillaries. In the capillaries, the protein-mediated transport pathway is predominant [110], and includes the activity of transporters, such as solute transporters (SLC transporters) and ATP-binding cassette transporters (ABC transporters). These transporters can generally be classified as transporters that passively diffuse lipophilic molecules, highly specific nutrient transporters, or transporters for the removal of waste products [34,111]. Typically, SLC transporters facilitate the uptake (influx) of molecules to the CNS, whereas ABC transporters are involved in the brain-to-blood (efflux) transport of molecules. As such, the transport of nutrients mediated by the SLC transporters is anticipated to play a key role in the development and functions of the brain via the ECs. In particular, SLC transporters on the surface of ECs assist with supplying the brain with the necessary energy and nutrients needed to function as they are facilitatory transporters responsible for the uptake of nutrients, amino acids, and small molecules, such as glucose, which is facilitated down the concentration gradient from the blood to the brain [111]. While SLC transporters are responsible for the uptake of nutrients, ABC transporters are ATP-driven, unidirectional efflux transporters that clear out metabolic waste and xenobiotics from the brain, and are critical for the defense system to clear out foreign substances [37,111]. In addition to the SLC and ABC transporters, transferrin receptor 1 (TfR1) (also known as transferrin receptor) is expressed on brain ECs, where it is crucial for iron transport into the brain and is also a target for drug delivery into the CNS. TfR1 is of special interest since its expression is limited to the brain ECs as opposed to peripheral ECs. We will discuss these transporters in brief, and the ethanol modulation of these transporters.

### 5.1. SLC Transporters

The SLC superfamily currently represents 52 families (SLC1 to SLC52) and 395 genes for individual transporters, and has been elegantly reviewed elsewhere [112]. Below we will discuss proteins from the SLC2 (facilitative GLUT transporter family) and SLC39 (metal ion transporter family) families due to the available data on ethanol’s effects on these transporters.

Glucose transporter type 1 (GLUT-1): The SLC2A1 gene codes for GLUT-1 of the sugar porter subfamily of the major facilitator superfamily [113]. The gene, located on the short arm of chromosome 1, is approximately 35 kb in length, with ten exons and nine introns [114]. While SLC2A1 is expressed in all human cells, erythrocytes and the cells forming blood-tissue barriers, such as the BBB and the blood-retinal barrier, show higher expression of the gene [115]. The amino acid sequence of GLUT-1 is highly conserved among species with 98% identity between humans and rats [116]. Mutations that disrupt the structure of GLUT-1 leading to the loss of function are lethal [113]. GLUT-1 is expressed in all cells and catalyzes facilitated diffusion of glucose into organs, including the brain, and provides the basal line of glucose supply for all cells [113,115]. It is also highly expressed in the placenta for the maternal–fetal transport of glucose during pregnancy [117]. Several types of cancer cells show overexpression of GLUT-1. Expression levels of GLUT-1 have a positive correlation with the proliferative index of tumor cells, with glucose being the primary energy source for tumor cells [113,118]. GLUT-1 of the BBB is responsible for the glucose supply for the brain, essential for brain energy metabolism. A defect in a SCL2A1 gene results in a rare genetic metabolic disorder called GLUT-1 deficiency syndrome (GLUT1-DS) [113,119]. GLUT1-DS has broad phenotypic symptoms and spectrums including epileptic seizures, movement disorders, cognitive behavioral impairment, hemiplegia of childhood, hemiplegic migraine -, cyclical vomiting, and stroke mimics [120].

Zrt- and Irt- like protein families (ZIP8): SLC39A8 gene codes for a ZIP8 metal cation transporter which is a member of the Zrt- and Irt- like protein family of metal transporters [121,122]. It is in chromosome 4 and is approximately 180 kb in length [123]. SLC39A is expressed in every cell type examined, localized in the plasma membrane and mitochondria, and shows high evolutionary conservation between human and mouse [124]. SLC39A(-/-) global knockout is lethal in mouse models. Human variants in SCL39A8 are associated with many developmental disorders, including hypomagnesemia, hypermanganesuria, and glycosylation deficiency [124]. ZIP8 is a transmembrane protein that mediates the transport of metal cations including zinc, manganese, iron, and cadmium into the cytoplasm. It shows a higher affinity to manganese than zinc in mammalian cells [121]. Hepatic ZIP8 regulates the metabolism of manganese in the liver, which then controls manganese homeostasis and the activity of manganese-dependent enzymes in the whole body [125]. Pulmonary ZIP8 functions as zinc transporters that protect lung epithelial tissues against inflammation and cytotoxicity [124]. The brain is the primary organ affected by the accumulation of manganese in the body. Manganese accumulation in the brain results in a condition called manganism which resembles Parkinson’s disease in its psychiatric and motor symptoms. In the brain, ZIP8 regulates the uptake and accumulation of manganese through the capillary endothelial tissues of the BBB [126]. How ZIP8 regulates manganese homeostasis and epithelial integrity is not well understood and requires further investigation.

In addition to the above SLC family proteins, the organic anion transporter (OAT) subfamily has gained considerable attention as it facilitates the uptake and excretion of various drugs and metabolites [127]. A significant part of the SLC22 (solute carrier 22) transporter family constitutes the OAT subfamily with the expression of the SLC22A6 gene in various tissues including kidney, liver, choroid plexus, olfactory mucosa, brain, retina, and placenta. Notably, the malfunction of these proteins is associated with the accumulation of toxic metabolites with reduced clearance, supporting their role in regulating metabolic diseases.

### 5.2. ABC Transporters

Based on amino acid sequence homology, the ABC transporter family has been categorized into seven main subtypes (ABCA–ABCG) and these transporters are involved in either the efflux or influx of endogenous and exogenous compounds [128]. Due to the high energy demands of the brain and the ATP required to drive ion gradients for ABC transporters, CNS ECs have 4- to 5- fold more mitochondria in the brain compared to ECs in other organ systems [36]. ABC transporters are selective, making them excellent barriers to pathogens and exogenous agents. However, this selectivity poses significant disadvantage in that, these transporters are unable to effectively distinguish between harmful toxins from therapeutic drugs, imposing a major obstacle in the development and delivery of therapeutic drugs for CNS disorders [129,130,131]. For example, the overexpression of ABCB1 (also known as P-glycoprotein (P-gp)) and ABCG2 in the brain ECs limits the penetration of certain exogenous compounds (drugs) into the brain, thereby limiting or abrogating their therapeutic efficacy [132,133]. P-gp is encoded by the ABCB1 gene in humans and the Abcb1a and Abcb1b gene isoforms in rodents. The regulation of P-gp is highly complex, affecting mRNA and protein levels as well as transporter activity. P-gp’s role in maintaining BBB integrity has been recognized [134]; however, its involvement in neurological, neuroinflammatory, and neurodegenerative conditions are relatively unexplored [135]. In this context, more attention has been given to exploring P-gp for its therapeutic benefit [136,137].

### 5.3. Transferrin Receptor 1 Transporter

Transferrin receptor 1 (TfR1) is a 97 kDa type 2 membrane-bound protein found in all vertebrates that mediates the endocytosis of iron-bound transferrin (Tf) complex. In humans, TfR1 is ubiquitously expressed in all cells, with higher expression in rapidly proliferating cells and energy-requiring cells such as cancer cells, osteoclasts, activated lymphocytes, and erythroblasts as it mediates the iron uptake of cells via the transferrin–transferrin receptor pathway [138,139]. It is essential for development in mammals, and mice that lack functional TfR1 are embryo-lethal due to impaired erythropoiesis and neurologic development [140]. TfR1 is partially responsible for the transport of iron across the BBB [141]. TfR1 has been studied as a therapeutic approach for CNS drug delivery through TfR1 mediated transcytosis pathways through the BBB [142].

### 5.4. Ethanol Interactions with Transporters

Data from human EC models demonstrates that a moderate to high concentration of ethanol inhibits GLUT-1 function and reduces the uptake and transport of glucose [105]. The study concluded that the reduction in GLUT-1 expression in the BBB was partially due to defective translocation of mRNA for the biosynthesis of GLUT-1, as cells treated with a neuroprotective agent with ethanol exposure did not show a reduction in the protein level [105]. In support of the in vitro findings, in vivo study using a chronic ethanol liquid-diet-model in rodents showed that the reduction in glucose uptake was correlated with the decrease in the expression of GLUT1 in the micro vessels of the BBB in the frontal, hippocampal, and occipital regions of the brain [105,143]. With respect to OATs, preclinical studies show that ethanol reduces the activity of OATs [144]. Such actions by ethanol may assist with the reduced clearance of alcohol metabolites, such as acetaldehyde, and contribute to toxicity. Genome-wide association studies associated the SLC39A8 gene in humans with alcohol use disorders, with nonsynonymous missense mutation in the gene associated with lower levels of alcoholic drinks per week [126,145]. Banna et al.’s study using mice models found no significant genotypic differences in alcohol consumption or preference between wild-type and SLC39A8 hypomorphic mice [145]. How SLC39A8 interacts with alcohol is unknown.

In vitro studies using human colon adenocarcinoma cell lines show that high concentrations of ethanol (>80 mM) significantly affect efflux transporters such as P-gp by increasing both mRNA and protein expression, with no observable difference in the function of P-gp [146]. Interestingly, withdrawal from ethanol normalized the levels indicating recovery from insult. Notably, the increases in P-gp were associated with increases in cytochrome P-450 enzymes [146]; however, the functional significance of this change in alcohol drinking and seeking are unexplored.

With respect to TfR1, in vitro studies using primary rat hepatocytes and liver biopsies from patients diagnosed with moderate to severe AUD show ethanol-induced increases in the expression of TfR1 [147]. Interestingly, increases in TfR1 were associated with ethanol-induced oxidative stress, supporting a mechanism for altered transporter function and the enhanced uptake of transferrin-bound iron into hepatocytes [147,148]. However, the behavioral significance of increased iron deposition due to increased TfR1 expression in the BBB in AUD is unexplored and requires further investigation. Taken together, emerging data from preclinical studies support ethanol’s multifaceted damage to endothelial cells, with binge or heavy ethanol experience impairing junctional complexes of endothelial linkage and acute ethanol experience disrupting the transporters of endothelial cells, therefore contributing to leakage of the BBB and compromising brain homeostasis.

## 6. EC Interactions with NVU Cell Types

CNS ECs also form partnerships with other cellular components, such as pericytes, mural cells, astrocytes and perivascular macrophages, and the glycocalyx to constitute the BBB’s NVU, giving rise to the highly selective and protective properties of the BBB [34]. In the following section we will discuss, in brief, interactions of ECs with each of these cell types and their significance in maintaining a healthy BBB. Lastly, we will briefly discuss ethanol modulation of pericytes, and perivascular macrophages and astrocytes.

### 6.1. ECs and Pericytes

Discovered in 1920 by Charles-Marie Benjamin Rouget, pericytes were described as a set of cells with contractile properties, surrounding ECs in small blood vessels. Since their discovery, many publications have examined pericytes, some drawing debates about their contractile properties [149]. These discrepancies may be partially explained by a lack of clear and consistent molecular markers, making it difficult to properly identify pericytes. As such, it has been a challenge to distinguish them from other mural cells (discussed below) that reside in the perivascular space, such as vascular smooth muscle cells [149]. There are many markers used to identify pericytes, including PDGFR-β, NG2, CD13, α-SMA, and desmin, however these markers are also expressed in other cell types [149]. A common strategy to study CNS pericytes is to co-label cells with PDGFR-β and NG2 [150]. Pericytes sit on the abluminal side of the basement membrane, extending long cytoplasmic processes in small blood vessels, including capillaries, venules, and precapillary arterioles [1,149]. Some works support Rouget’s original findings, suggesting that pericytes have contractile properties and contribute to regulating cerebral blood flow [151,152,153]. However, this remains a point of contention, with some papers instead citing vascular smooth muscle cells as primary contributors to flow regulation [154,155]. As previously mentioned, these discrepancies may be partially explained by the lack of consistent molecular markers for pericytes. 

Pericytes contribute to BBB integrity by regulating endothelial transcytosis, maintaining capillary blood flow, and TJ formation, with pericyte-deficient mice demonstrating increased BBB permeability [156,157,158]. Sharing a basement membrane, ECs and pericytes reside in close proximity to one another. However, ECs and pericytes form connections at select points, due to holes in the basement membrane. Through a peg-socket connection, pericytes’ cytoplasmic “fingers” anchor into the endothelial apertures [149]. EC-pericyte adhesion and signaling occurs via N-cadherin, an adherens junction protein [159]. It has been suggested that pericytes provide signaling connections between vascular and neuronal components of the NVU, demonstrated through GJ coupling, although there is much controversy on its exact role in neurovascular coupling [154,160]. With varying opinions on the degree of pericytes’ contractile properties, to fully understand its properties and functions in the context of cerebral blood flow requires future investigation.

### 6.2. ECs and Perivascular Macrophages

Perivascular macrophages (PVMs) are a distinct type of innate immune myeloid cells that are distributed across the endothelial lining of blood vessels in various organs, playing multifaceted roles in maintaining vascular homeostasis [161]. In the CNS, they maintain the integrity of the BBB under physiological conditions, while also being involved in intricate mechanisms contributing to the pathogenesis of neurological diseases [162]. It was once believed that the origin of PVMs was the same as other peripheral tissue macrophages, which originate from the mesoderm, but recent findings suggest that PVMs arise at E10.5 from early erythromyeloid precursor cells located in the yolk sac [163,164]. Staining using CD206 antibodies has revealed that PVMs have an elongated strip-like shape that is distributed along the fluid-filled perivascular space along cerebral arteries and venules [162]. The perivascular space in the brain is confined by the vascular basement membrane on the abluminal side and the glia limitans basement membrane on the side facing the brain tissue. It is sealed off at small arterioles and capillaries (<10 μM) where the two membranes merge [165]. Situated in the Virchow–Robin space, PVMs neighbor ECs separated by the basement membrane [166]. This strategic position at the interface between the brain tissue and the blood vessels allows PVMs to act as mediators between the peripheral immune system and the brain’s immune environment, providing structural and functional support for the BBB. It is thought that PVMs associate with endothelial junctions, communicating with ECs through the release of pro-inflammatory cytokines and chemokines [167]. However, the functional significance of PVMs in the health and plasticity of ECs is unclear and requires further investigation.

### 6.3. ECs and Astrocytes

Astrocytes provide support to the NVU through the formation of perivascular astrocytic endfeet, which ensheathe ECs and pericytes, and relay signals between neurons and ECs that help regulate blood flow [168,169]. Astrocytes are also a major source of sonic hedgehog (SHH) signaling in the brain which is important in the expression of several pathways and genes which are important for maintaining the BBB ECs’ function [170]. The neutralization of SHH signaling in primary human cell cultures and in the CNS of SHH^-/-^ embryos resulted in the decreased expression of major EC proteins, such as JAM-A, Cdh5, claudin-3, and claudin-5 [170]. In vivo and in vitro SHH activation in ECs was also found to decrease the expression of chemokines such as CXCL8 and CCL2 cellular adhesion molecule-1, and pro-inflammatory T helper cells T_H_1 and T_H_17, suggesting its role as an anti-inflammatory modulator of the NVU [170].

### 6.4. ECs and Mural Cells

Mural cells include pericytes and vascular smooth muscle cells which surround the endothelial monolayer. Smooth muscle cells are present in large vessels, completely surrounding the monolayer, distinct from pericytes which incompletely surround the endothelium in capillaries [154]. Smooth muscle cells are responsible for cerebral blood flow through a process called neurovascular coupling, with the endothelium releasing vasoactive factors such as nitric oxide and prostacyclin to manipulate vascular muscle cells [154,155].

### 6.5. ECs and Glycocalyx

Evenly distributed on the luminal surface of ECs, glycocalyx is a layer composed of glycoproteins, glycosaminoglycans, and proteoglycans, essential for BBB homeostasis [171,172]. They are synthesized and secreted by ECs, binding to the surface with glycoproteins and proteoglycans. Glycoproteins consist of integrin receptors and immunoglobulin proteins which mediate EC adhesion, through molecules such as intercellular adhesion molecule 1 and 2, vascular adhesion molecule 1, and platelet/endothelial cell adhesion molecule 1 [173]. Not only does glycocalyx maintain EC adhesion, but it also provides a negative charge to the BBB surface, so degradation or neutralizing glycocalyx results in increased BBB permeability [173].

### 6.6. Molecular and Cellular Effects of Ethanol on Pericytes

The molecular and cellular effects of ethanol on pericytes represent a critically understudied area of alcohol research, despite their essential role in BBB integrity and microvascular function. Given the multifaceted role of pericytes within the brain, future studies need to characterize the cellular and molecular effects of ethanol on pericytes. One such study by Vore et al. shows that the cellular marker for pericytes, *Pdgfrb*, showed an increase in immunofluorescence after adolescent intermittent ethanol exposure [174]. In addition, ex vivo pericytes from the brain of three-week-old rats were treated with 50 mM of ethanol and demonstrated that the mRNA of genes involved with innate immune activation such as *Tlr4* and *Nlrp3* and cytokines such as increased *Il1b* were increased after ethanol treatment; however, protein levels were not changed [175]. This discrepancy in mRNA and protein levels could be attributed to the length of exposure and age of the rats at the time of pericyte isolation. Taken together, these two studies provide the rationale that pericytes are responsive to ethanol and could contribute to BBB integrity dysfunction after ethanol consumption, potentially through innate immune activation.

### 6.7. Ethanol Interactions with PVMs and Astrocytes

One of the central mechanisms underlying alcohol-induced neurovascular damage involves the infiltration and activation of monocyte-derived macrophages, particularly PVMs and is largely mediated by inflammatory and oxidative stress pathways [176]. PVMs act as key immune sentinels, responding to pathological insults and maintaining cerebrovascular homeostasis. However, chronic alcohol exposure disrupts this equilibrium by inducing a pro-inflammatory phenotype in these cells, characterized by the increased expression of cytokines such as tumor necrosis factor-alpha (TNF-α), interleukin-6 (IL-6), and monocyte chemoattractant protein-1 (MCP-1), which further exacerbate BBB permeability [177]. Additionally, alcohol-induced oxidative stress plays a pivotal role in impairing the function of PVMs. Chronic ethanol consumption enhances the production of reactive oxygen species and nitric oxide, leading to mitochondrial dysfunction and exacerbated lipid peroxidation within these immune cells [178]. This oxidative environment not only accelerates endothelial damage but also dysregulates PVM signaling, further potentiating neuroinflammatory cascades [179]. In parallel, in vitro studies demonstrate that ethanol increases the secretion of pro-inflammatory molecules by astrocytes. Mechanistically, ethanol-induced activation of cytosolic phospholipase A2 and cyclooxygenase-2 via interaction with the activity of toll-like receptor 4 and Src kinase in astrocytes increased the secretion of inflammatory signals [180]. Taken together, these studies support the direct effects of ethanol on astrocytes and the indirect effect of ethanol on the NVU via altering astrocyte signaling.

## 7. Sex-Dependent Regulation of the BBB: Potential for Regulation of Ethanol Consumption

Findings from clinical studies demonstrate significant gender differences in the neurotoxic effects and cognitive impairing effects of alcohol in individuals suffering from mild to severe AUD [181,182,183], with women being more vulnerable to the effects of alcohol and men consuming alcohol at higher levels compared with women [184,185]. Somewhat supporting the clinical findings, animal models of moderate to severe AUD show that female rodents self-administer more ethanol in an operant paradigm compared to male rodents [186]. Even though drinking data appears to be somewhat different than that reported in the human condition, it is notable that clinical research on the transition to alcohol dependence report that once women begin drinking regularly they progress faster than men to drinking-related problems [187,188,189], supporting the clinical significance of the increases in ethanol drinking in female rats under ethanol vapor-induced dependent conditions and during relapse compared with males [18].

Transcriptomic analysis has revealed the brain’s vasculature is highly heterogenous [190,191,192,193]; however, limited studies have characterized the molecular and cellular differences between male and female microvasculature of the BBB. A handful of studies have directly characterized the molecular underpinnings of the BBB between males and females [194,195,196,197,198,199]. Given that ethanol effects the BBB permeability in males and females differently, with males showing greater increases in permeability to binge ethanol exposure [200], little is known about how these baseline differences could drive ethanol consumption or contribute to BBB integrity. Mechanistically, active alcohol metabolizing enzymes [201], gonadal hormones [202], influence of gonadal hormones on dopaminergic neurotransmission [203] or sexual dimorphism in the mitochondrial metabolic protein profiles of microvessels [197] could be factors contributing to ethanol’s sex-specific cerebrovascular and neurological pathologies. For example, female microvessels have an enrichment of genes related to mitochondrial function and ribosome biogenesis while males have an abundance of genes regulating nucleic acid binding, transcription factors, PI3K pathway, and amino acid transporters [195,196,197,198,199]. More notable is that female rats have higher levels of arterial mitochondrial bioenergetics compared with males [197], suggesting resistance to alcohol-induced disruption of cellular energy metabolism and tissue injury [204]. The aim of this section is to highlight genes that are differentially expressed between male and female within the BBB and may mechanistically contribute to observed sex differences in ethanol consumption leading to barrier integrity dysfunction [18].

Slc22a8: Slc22a8 (OAT3) is an organic anion exchanger located on the abluminal (brain side) of ECs of the BBB. This gene has several critical functions for maintaining the BBB such as the exchange of glutarate and ketoglutarate [205], the drug efflux of toxic drug metabolites from the brain to the blood [206], a substrate for several antiviral drugs [207], and is enriched in the ECs of the BBB [192] and in the choroid plexus [207,208]. In fact, the global knockout of Slc22a8 in the choroid plexus showed decreased fluorescence uptake of Dextrans, suggesting it is important for transcellular transport [208]. In the case of alcohol exposure, Slc22a8 has been shown to be down-regulated during chronic intermittent ethanol exposure in the male rat central nucleus of the amygdala [209] and after ethanol withdrawal in the hippocampus [210]. Since this gene is the main regulator of drug efflux, further understanding of this gene is critical to understand how ethanol can contribute to BBB drug efflux and the removal of waste metabolites in the brain.

Cd9*:* Another gene enriched in male vasculature is *Cd9*. *Cd9*, a membrane-bound tetraspanin protein which is associated with integrins on the cell surface [211]. In addition, *Cd9* is expressed throughout several BBB cell types including ECs in vitro [212] and in vivo [213], and vascular smooth muscle cells in vitro [214,215] and in vivo [213,216]. Furthermore, cell type-specific expression of *Cd9* in ECs has been linked to the modulation of neuroinflammation by contributing to leukocyte migration during inflammation [217]. Interestingly, recent transcriptomic studies have identified *Cd9* as being responsive to the effects of ethanol [209,210,218], suggesting that Cd9 could be a potential target for the neuroimmune effects of alcohol consumption through modulating the sex-dependent modulation of BBB. Taken together, Cd9 could be a potential therapeutic target for rescuing the effects of BBB loss after chronic ethanol consumption.

Src*:* Src encodes for tyrosine protein kinase and is involved with several cell growth and other cellular functions. The Src gene is enriched in male micro vessels [195] and has been shown to be expressed in the ECs [219] and pericytes [220] of the BBB. Mutations in the Serine 75 residue of Src suggests that it increases ethanol consumption and preference [221]. In utero exposure to ethanol induces a robust induction of Src [222]; however, this study did not address its effect on ECs and/or pericytes. There is evidence which suggests that Src could have contractile properties on arteries [223]. Despite this data from cerebral arteries, further work addressing pericytes and smooth muscle cells could also play a role in the contractile effect on cerebral arteries or capillaries after ethanol consumption. Taken together, Src could be an interesting target for understanding other modalities of ethanol consumption through neurovascular effects.

Igf1r*:* Igf1r is in the family of membrane-bound tyrosine kinases with high expression in ECs. Igf1r is critical for various vascular-specific functions such as angiogenesis [224], maintenance of the BBB [224,225,226,227,228], and neuroinflammation [226]. The ligand for Igf1r is IGF-1 with a six-to-eight-fold higher affinity than other insulin peptides [229]. The teratogenic effects of ethanol contribute to Igf1r dysfunction with the evidence primarily in neurons [229,230,231,232,233,234]. Chronic voluntary ethanol consumption results in the reduced binding of both insulin and Igf1 in the temporal lobe, hypothalamus, and cerebellum in adult rats [230]. In addition, a recent study showed that Igf1r was down-regulated in the male prefrontal cortex after chronic ethanol exposure, but up-regulated in the female prefrontal cortex, suggesting a sexual dimorphic role of potential BBB protection in females [235]. The data suggests that ethanol can contribute to NVU dysfunction; however, there are no causal experiments demonstrating that effect.

Ribosomal genes: Several Ribosome-specific genes have consistently been shown to be enriched in female micro vessels [194,195,196,226] and arteries [167]. Several large and small subunits including RPS26, RPS21, RPL10, and RPSA have been shown to be enriched in female brain micro vessels [195,199]. The structural small surface-associated ribosomal protein (RPSA) gene has been associated with BBB function [236,237,238,239,240]. Also, RPSA is suggested to play a role in several neuroinflammatory conditions by interacting with viral nucleic acids including HSV-1 viruses [241] and bacteria [242]. In addition, ethanol exposure in zebrafish reduces the expression of RPSA [243], and in rodents and humans reduces brain and blood levels of RPSA [235,244], suggesting that RPSA is negatively regulated by ethanol exposure. Further studies are needed to better understand how RPSA, ethanol, and its interaction with the BBB could contribute to female-dependent regulation of the microvasculature.

## 8. Future Directions of Ethanol and the BBB

Given the complexity of the BBB in cellular composition, molecular makeup, and sex-dependent contributions, there remain several open areas for future research directions. For instance, it is well-established that the genetic background of mice, for instance, results in differences in ethanol consumption [245,246] as well as could contribute to brain regional differences in BBB permeability [198]. However, the link addressing the role of ethanol, genetic background, and BBB permeability remains unknown. Other open questions also encompass the use of more in vivo models of the BBB and regulation of ethanol exposure and consumption. Finally, although transcriptomic and proteomic studies have identified different mechanisms governing BBB integrity and function, the sex-specific effects of ethanol on the molecular regulation of BBB permeability, specifically EC function, are still not understood. Given that BBB permeability using fluorescently labeled tracers shows consistently that females are less permeable than males [247,248], future studies should address a causal interaction with EC function, ethanol, and sex-dependent factors.

## Data Availability

No new data were created or analyzed in this study. Data sharing is not applicable to this article.
